# Hepatitis C Virus P7—A Viroporin Crucial for Virus Assembly and an Emerging Target for Antiviral Therapy

**DOI:** 10.3390/v2092078

**Published:** 2010-09-27

**Authors:** Eike Steinmann, Thomas Pietschmann

**Affiliations:** TWINCORE†, Division of Experimental Virology, Centre for Experimental and Clinical Infection Research, Feodor-Lynen-Str. 7, 30625 Hannover, Germany; E-Mail: eike.steinmann@twincore.de

**Keywords:** HCV, p7, assembly and release, ion channel, viroporins, antiviral therapy

## Abstract

The hepatitis C virus (HCV), a hepatotropic plus-strand RNA virus of the family *Flaviviridae*, encodes a set of 10 viral proteins. These viral factors act in concert with host proteins to mediate virus entry, and to coordinate RNA replication and virus production. Recent evidence has highlighted the complexity of HCV assembly, which not only involves viral structural proteins but also relies on host factors important for lipoprotein synthesis, and a number of viral assembly co-factors. The latter include the integral membrane protein p7, which oligomerizes and forms cation-selective pores. Based on these properties, p7 was included into the family of viroporins comprising viral proteins from multiple virus families which share the ability to manipulate membrane permeability for ions and to facilitate virus production. Although the precise mechanism as to how p7 and its ion channel function contributes to virus production is still elusive, recent structural and functional studies have revealed a number of intriguing new facets that should guide future efforts to dissect the role and function of p7 in the viral replication cycle. Moreover, a number of small molecules that inhibit production of HCV particles, presumably via interference with p7 function, have been reported. These compounds should not only be instrumental in increasing our understanding of p7 function, but may, in the future, merit further clinical development to ultimately optimize HCV-specific antiviral treatments.

## 1. Introduction

Hepatitis C virus (HCV) infection is a major cause of chronic liver disease. Currently about 130 million individuals are persistently infected with HCV [1]. In the course of two to three decades, ongoing virus replication puts these patients at risk to develop liver cirrhosis and hepatocellular carcinoma [2]. In fact, HCV infection is the most frequent indication for liver transplantation [3]. HCV is an extraordinary variable positive strand RNA virus belonging to the genus *Hepacivirus* within the family *Flaviviridae*. Patient isolates are grouped into at least six major genotypes deviating from each in as much as 30% of the nucleotide sequence [4]. At present, neither a selective antiviral therapy nor a vaccine for HCV is available and the only current therapy, a combination of pegylated Interferon-α and Ribavirin, is associated with severe side effects and is efficacious in only 50% of patients infected with the most prevalent viral genotype 1 [5]. Clinical trials of novel enzyme inhibitors have revealed that drug-resistance mutations compromise the efficacy of these antiviral compounds in monotherapy [6]. Moreover, for many drugs in development, narrow genotype-specificity limit their utility for treatment of patients infected with varying viral strains. Therefore, novel substances with antiviral activity, different mode of action, and ideally cross-genotype efficacy, are urgently needed to implement highly active combination treatment regiments for control of virus replication and escape.

**Figure 1.**
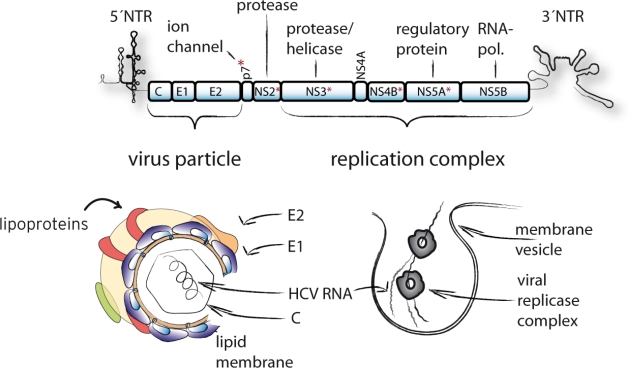
Schematic representation of the HCV genome, virus particle and membrane associated replication complex. Note: The virus particle is composed of the viral structural proteins core, envelope protein 1 and 2, as well as host cell lipoproteins. Whether additional viral proteins are incorporated (e.g., p7) is presently unknown. The minimal viral factors needed for establishment of functional replication complexes are the NS3 to NS5B proteins [12]. Viral proteins that have essential co-factor function during virus assembly are highlighted by red asterisks.

The HCV genome of about 9.6 kb is composed of the 5′ non-translated region (NTR), an open reading frame encoding a large polyprotein, and the 3′ NTR [7] (Figure 1).

A set of cellular and two viral proteases, NS2/3 and NS3/4A, liberate the individual viral proteins. The N-terminal portion of the polyprotein contains the structural proteins core, envelope glycoprotein 1 and 2 (E1, E2) that constitute the virus particle. These proteins are cleaved from the polyprotein by the host cell signal peptidase [8,9]. In case of the core protein, an additional cleavage step mediated by the signal peptide peptidase liberates the mature C-terminus [10]. Further downstream the polyprotein harbors p7, a short integral membrane protein, and the non-structural (NS) proteins NS2, NS3, NS4A, NS4B, NS5A and NS5B. Proteins NS3 to 5B are the minimal viral components of the membrane-bound replication complexes which catalyze RNA replication [11,12].

For years, HCV researchers have struggled with the lack of adequate tissue culture systems sustaining virus replication and thus permitting molecular analyses of HCV replication. However, recent development of novel cell culture models, including subgenomic replicons [12], HCV pseudoparticles [13,14], and ultimately the JFH1-based infection system [15–17], has not only gradually removed this roadblock, but also lead to the accumulation of new insights into essential aspects of the HCV replication cycle [18]. With regard to assembly and release of infectious HCV particles, essential roles of viral and host factors have been defined. On one hand, cellular factors involved in the secretion of lipoproteins like the microsomal triglyceride transfer protein (MTP), apolipoprotein B and E (ApoB, ApoE), have been recognized as essential co-factors of HCV particle production [19–21]. On the other hand, it was realized that besides the canonical structural proteins core, E1 and E2, also other viral factors including NS5A, NS4B, NS3, NS2 and p7, play crucial, yet at present poorly understood, roles in the production of infectious viral progeny [22–26].

These novel findings highlight the complexity of the HCV assembly process which is coordinated by multiple viral and host-derived factors and is tightly linked with the cellular lipoprotein secretion pathway. Understanding and dissecting the individual contributions of these factors will be a major challenge that will engage molecular virologists and cell biologists in the future. At the same time these studies are likely to reveal novel targets and modes for antiviral therapy. In this regard, the smallest among the viral assembly co-factors, p7, has recently attracted a lot of attention. Here we review current knowledge of p7 structure and function and highlight possible avenues for p7-based inhibition of HCV replication.

## 2. Structure and Function of HCV P7

### 2.1. P7 Topology and Structure

The HCV p7 protein is a small, integral membrane protein of 63 amino acids that is encoded at the junction between the structural and non-structural region of the HCV polyprotein. Although most cleavages in the HCV polyprotein precursor proceed to completion during or immediately after translation, cleavages are delayed at the E2-p7 and p7-NS2 sites, leading to the production of an E2-p7-NS2 precursor [27]. In addition, processing between E2 and p7 is incomplete resulting in the production of fully processed E2 and uncleaved E2-p7 [28,29].

When expressed on its own in mammalian cells, epitope tagged p7 has been shown to insert into ER membrane with two membrane-spanning helical domains, both its N- and C-termini exposed to the ER lumen, and a short hydrophilic loop facing the cytosol [30]. Alternatively, a protein topology where the C-terminus is orientated towards the cytoplasm may be possible [31]. The second transmembrane helix of p7 acts as a signal peptide to target NS2 to the ER, although when expressed by itself, NS2 is able to insert into membranes also independent of any p7 sequence [32].

**Figure 2.**
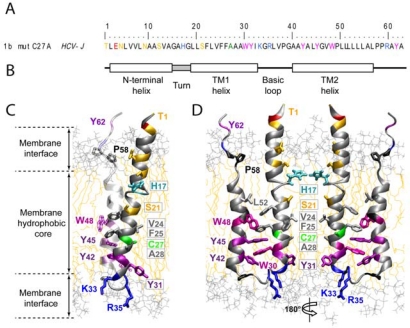

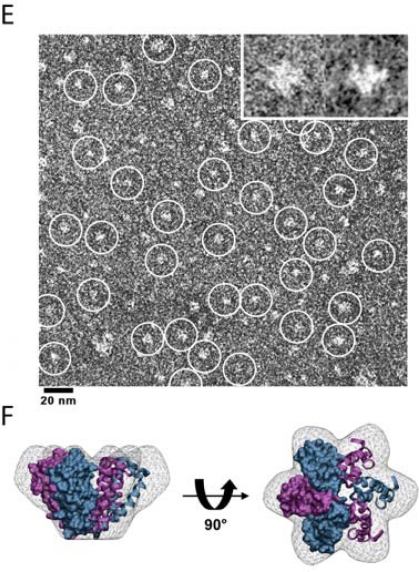
Structure of monomeric and hexameric HCV p7. Note: **(A)** Primary sequence of HCV-J p7 protein (genotype 1b) with color code as follows: *Yellow*, Ser, Thr, and Asn; *green*, Cys; *cyan*, His; *blue*, Arg and Lys; *red*, Glu; *magenta*, Trp; *purple*, Tyr; *black*, Pro; *gray*, any other residues. **(B)** Schematic representation of helical, turn, and loop regions deduced from the NMR structure [37] and **(C, D)** side view of the p7 representative monomer. Residues which are expected to point towards the ion channel are highlighted with boxes. **(E)** Single particle electron microscopy of the HCV p7 channel as recently reported by Luik *et. al.* [36]. A typical view from a raw image of p7 oligomers negatively stained with phosphotungstic acid at low magnification is given. **(F)** The authors fitted simulated p7 monomers into the hexameric volume of the structure derived by electron microscopy. C and N termini of the atomistic p7 model are oriented toward the petal tips. **(A–D)** are reprinted from [37] and **(E)**, **(F)** from [36] with permission.

Early electron microscopy studies suggested that p7 monomers assemble into hexamers [33], or heptamers [34] in artificial membranes. Computational modeling and secondary structure predictions were used to create structural models of monomeric and oligomeric p7 [30,35]. Recently, the three-dimensional structure of hexameric p7-complex was determined by single-particle electron microscopy at a 16 Å resolution [36]. Chemically synthesized full length p7 monomers were detergent solubilized, negatively stained, imaged using the random conical tilting approach and analyzed by single particle reconstruction. Using this approach, a hexameric complex with a flower-shaped architecture and six protruding petals oriented toward the ER-lumen was revealed [36] (Figure 2). This report of the entire p7-protein complex provides detailed molecular insights into p7 structure and function and will facilitate drug design in the future. Very recently a detailed structural analysis of monomeric p7 has been reported [37]. Using NMR and molecular dynamics simulation, Montserret *et al.* observed an unexpected N-terminal α-helix which is connected to the first transmembrane helix (TM1) via a short turn. In addition, a long cytosolic loop extending from residue 33 to 39, including the di-basic motif and connecting TM1 and TM2 was revealed [37]. Importantly, some of these observed structural elements, for instance the N-terminal α-helix or a longer cytosolic loop, differ from previous predictions of the secondary structure of p7 [37].

### 2.2. P7—A Viroporin and True Ion Channel?

*In vitro*, p7 forms oligomers and is capable of conducting ions across artificial membranes in a cation-selective manner [33,37–39]. Consistent with the structure of p7, electrophysiological studies indicate that the N-terminal helix of p7 is oriented towards the channel pore [40,41]. Due to the ability to sustain ion fluxes, p7 has been included into the expanding family of viral proteins known as viroporins. Viroporins are small, virus-encoded polypeptides that interact with membranes comprising at least one transmembrane segment. Moreover, oligomerization is an essential prerequisite for their ability to modify the membrane’s permeability to ions or other small molecules [42]. Typically, viroporins are small hydrophobic proteins comprised of about 60–120 amino acids that encode at least one transmembrane passage. The family includes viral proteins from diverse virus families including 6k of alphaviruses, M2 of influenza virus A, vpu of HIV-1 or picornavirus 2B. Generally, viroporins are not essential for RNA replication of the virus, but their presence promotes virus assembly/release and, in the case of the M2 protein, also virus entry. Interestingly, it was recently shown that the human polyoma JC virus, a non-enveloped DNA virus, also encodes a viroporin: The JC virus agnoprotein forms homo-oligomers, permeabilizes membranes and promotes virus release [43]. However, with the exception of influenza A virus M2 and HIV-1 vpu, little is known as to how these viral factors contribute to the release of infectious particles. Moreover, the rather broad definition of viroporins includes proteins which rather unspecifically permeabilize membranes and, at the same time, true ion channels with precise ion selectivity and intricate gating mechanism. Given these fundamental differences, it is likely that individual viroporin family members facilitate virus production by different mechanisms. Our mechanistic understanding of the role and function of a viroporin, and a direct link between its ability to conduct ions and its role in the virus life cycle, is probably most refined for the influenza A virus M2 protein. M2 is a proton-specific channel that is gated by pH [44,45]. Besides a crucial role of this protein during virus entry and uncoating, the proton channel function is important to prevent acidification of the *trans*-Golgi network thus avoiding premature conformational changes of influenza hemaglutinin in virus-producing cells [46].

Recent evidence suggests that the ion channel activity of HCV p7 (genotype 1b) is activated at acidic pH [47]. Furthermore, p7 was shown to substitute for M2 in facilitating the transport of the influenza HA protein to the cell surface in a cell-based assay [48]. Together these results imply that HCV p7 may operate in an M2-like fashion to facilitate HCV particle production. Both influenza A virus and HCV carry viral fusion proteins who’s fusion activities are triggered by conformational changes induced by low pH [14,49,50]. Therefore, dissipating the low pH of cellular secretory compartments may protect the HCV glycoproteins from premature induction of membrane fusion by low pH, thus facilitating production of infectious viral progeny. However, secreted HCV particles are resistant to low pH and, only after prolonged incubation of cell surface-bound particles at 37 °C, the virus acquires increased pH-responsiveness which then permits triggering of virus cell fusion [50]. This indicates that secreted HCV particles require certain additional stimuli like for instance receptor interactions to prepare the virus for low pH-induced fusion and infection. Although these findings make a role of p7 in protecting glycoproteins from low pH during assembly appear less likely, it is important to realize that such a function of p7 could also be crucial to allow proper folding of the glycoproteins. After all it is possible that immature glyocoprotein complexes or more generally viral assembly intermediates are susceptible to low pH-dependent misfolding. As a consequence, dissipation of low pH in the secretory compartments by p7 may be an essential prerequisite for production of infectious viral progeny.

In case of Influenza virus M2, channel opening is induced by protonation of the so called “pH-sensor” histidine at position 37 at low pH [51]. Recently, a histidine of p7 at position 17 has been suggested to function in a homologous fashion [47,52]. However, this position is not fully conserved among p7 proteins from different HCV genotypes (Figure 2 and [53]). Moreover, mutation of this residue abrogated ion channel function of genotype 1b-derived p7 [47] while the same mutant in the context of a genotype 1a-derived p7 exhibited wild-type ion channel activity [40]. Recently, Meshkat *et al.* analyzed the relevance of p7’s HXXXY/W motif (position 17–21) by reverse genetics using the JFH1 infection system. However, mutation of His-17 slightly increased production of infectious particles [54] indicating that this amino acid is not absolutely essential for p7 function. Although these results cast some doubts on the relevance of His-17 as essential residue for gating of p7 ion channeling, the notion that HCV p7 may act as a proton channel that is crucial for virus assembly certainly remains an interesting hypothesis that warrants further analysis. The recent progress made in the structural analysis of p7 should facilitate and guide these experiments, thus ultimately increasing our understanding of the p7 ion channel. In fact, the recent NMR structure of a genotype 1b p7 monomer (Figure 2) suggests that several hydrophobic residues protrude towards the channel lumen (e.g., V24 and F25) [37]. In the context of a p7 oligomer these residues may form two rings of hydrophobic side chains which could act as an energetic barrier limiting the permeability of the channel towards ions. Conformational changes of the monomers may move these residues apart, thus opening the channel for conductance of ions. Although more experimental work is necessary to confirm this hypothesis, the aforementioned rings of hydrophobic amino acids are reminiscent of those observed in channels like the nicotinic acetylcholine receptor [55], the MscL channel [56] and the bacterial K_channel [57] where rigid movements of helical channel segments permit channel opening and thus ion conductance.

### 2.3. Subcellular Localization of P7

A number of studies have investigated the subcellular localization of p7 in transfected cells as the partitioning of p7 to individual cellular compartments may provide important clues about its function. However, due to the paucity of useful p7-specific antibodies, mostly epitope-tagged p7 variants have been employed and localization studies of virus-producing cells with functional p7 are still lacking. Carrere-Kremer *et al.* reported that ectopically expressed p7 mainly localized to the endoplasmic reticulum of HepG2 cells while a small percentage of the over-expressed p7 protein was detected at the plasma membrane [30]. Griffin *et al.* utilized p7-GFP or FLAG-p7 fusion constructs and observed co-localization of p7 with mitochondrial-associated ER and mitochondrial membranes in 293T cells [48]. This pattern was also seen when p7 was expressed in the context of other HCV structural proteins. In fact, epitope-labeling p7 at its C-terminus primarily revealed an endoplasmic reticulum distribution whereas labeling the N-terminus yielded mostly a mitochondria-like localization [58]. Given these findings, these authors suggested that separate pools of p7 resident in distinct cellular compartments and playing specific roles in the viral replication cycle may exist. With the development of an antibody to the C-terminus of genotype 1b-derived p7, an ER-like localization of native untagged p7, independent of the presence or absence of the upstream signal peptide, was confirmed in transfected 293T [58]. More recently, localization of epitope-tagged p7 proteins expressed in the context of replication competent JFH1-based full length genomes were reported [59]. This study confirmed that also enhanced green fluorescent protein (eGFP)- or a hemagglutinin (HA)-tagged p7 localizes to the ER in transfected human hepatocarcinoma cells (Huh-7) [59]. However, unfortunately the GFP-tagged recombinant genome was not assembly-competent and the HA–tag in the JFH1 genome reverted to the wild-type sequence. Since both tagged p7 variants did not sustain production of infectious particles, further studies are necessary to establish localization of functional p7 in virus producing cells. Nevertheless, the important findings summarized above are consistent with a model where p7 modulates the cellular secretory compartments, most notably the endoplasmic reticulum. As the luminal pH of the secretory pathway is a key determinant of post-translational modification, protein and lipid sorting (the interested reader is referred to [60]), it is conceivable that modification of luminal milieu may be important for virus production. Furthermore, the localization studies described above with over-expressed p7 constructs, suggest that p7 may also modify mitochondrial functions.

### 2.4. Clues of P7 Function Based on Animal and Tissue Culture Experiments

The first proof for an essential function of p7 came from animal experiments, which established that recombinant genomes encoding mutant p7 proteins did not initiate productive infection upon intrahepatic injection into chimpanzees [61]. Moreover, disruption of uncleaved E2-p7 or p7-NS2 by insertion of an encephalomyocarditis virus (EMCV) internal ribosomal entry site (IRES) and mutations of the conserved di-basic motif within the cytoplasmic loop, abolished infectivity in this system. However, it was unclear how mechanistically p7 contributes to HCV infection.

At this time, experiments with subgenomic HCV replicons (which do not encode p7) in tissue culture had already confirmed that p7 was dispensable for HCV RNA replication [12], thus indicating that p7 functions in life cycle steps that are not modeled with subgenomic replicons. When Bartosch and Hsu reported that infectious HCV pseudoparticles, *i.e.* retroviral particles carrying HCV glycoproteins in place of retroviral envelope proteins, are readily produced in 293T cells and infect human liver cells in envelope protein- and receptor-dependent fashion, it was clear that also key steps of viral entry occur independent of p7 [13,14]. Recent work applying the JFH1-based infection system has now firmly established that p7 is required for assembly and release of infectious HCV particles [53,62].

Transfection of virus genomes carrying partial or complete deletion of p7 or different point mutations reduced or ablated production of infectious particles [53,62]. These genetic studies also highlighted an important role of the fully conserved di-basic motif for p7 function which at the same time is also crucial for ion channeling in artificial membranes [48]. Of note, replacement of the di-basic motif in p7 (residues K33, R35) of the efficient genotype 2a chimera Jc1 by two glutamine residues (Q33, Q35) reduced not only production of total infectivity by *ca*. 100-fold. At the same time this mutation also profoundly changed the ratio between cell-associated infectious particles (which are released upon rupturing the cells by consecutive cycles of freeze and thaw) and released particles [53]. These results suggest that p7 is not only important for assembly, but also for efficient release of infectious progeny from cells. The specific infectivity of these particles (*i.e.* the infectivity per given number of physical particles), however, was not reduced compared to wildtype particles. This observation provides some indirect evidence supporting the notion that p7 is dispensable for virus entry, since if p7 was important during entry, it is unlikely that particles produced with partially defective p7 are as infectious as wild-type HCV [53]. Further clarification of this issue awaits the precise proteomic analysis of HCV particles which should answer the question if p7 is incorporated into mature viral particles and in turn modulates their properties. It is worth mentioning here that the p7 protein of bovine viral diarrhea virus (BVDV), a relative of HCV from the genus pestivirus of the family *flaviviridae*, is also essential for the production of infectious particles [63]. However, BVDV p7 was not found in secreted virus particles, suggesting that BVDV does not package p7 into mature virions [64].

Tissue culture experiments have revealed additional features of p7 that should help to understand its function and mode of action: First, recent evidence indicates that p7 is absolutely essential for production of infectious HCV particles [53,65]. This feature separates p7 from other viroporins, which only facilitate assembly and release of infectious progeny, and may be a reflection of a unique mode of action. At first, however, it was unclear if solely defective p7 was responsible for ablation of virus production or if alternatively aberrant polyprotein processing caused by mutation of p7 was accountable as well. P7 harbors a signal sequence for insertion of NS2 into the ER lumen and processing of the E2-p7 and p7-NS2 signal peptidase cleavage sites is precisely regulated by E2, NS2 and notably also p7 sequences adjacent to the cleavage sites [30,66]. In fact, some p7 mutations like exchange of the di-basic motif for hydrophobic alanine residues, or deletion of p7 in the polyprotein, have a substantial impact on polyprotein processing, thus complicating interpretation of these data. Using a trans-complementation system where p7-defective full length genomes are rescued by HCV replicons expressing p7 *in trans*, Brohm *et al.* recently resolved this issue [65]. Although partial deletion of p7 in the polyprotein slightly changes polyprotein processing, virus production of this construct was restored when only p7 was expressed from helper replicon *in trans*. This finding not only firmly established the essential role of p7 for virus production, it also now provides a simple model to further investigate p7 function in the absence of secondary effects on polyprotein processing.

Second, p7 function cannot be replaced by viroporins from other viruses. At least the two viroporins analyzed so far, influenza A virus M2 and HIV-1 vpu, were unable to rescue assembly of p7-defective full length genomes [65], arguing that p7 exerts an HCV-specific function.

Third, a number of genetic evidences now strongly suggest that p7 acts in concert with additional viral factors, which may in part explain, why M2 and vpu are unable to compensate for p7: Haqshenas *et al.* reported that an inter-genotypic JFH1 chimera with GT1b p7 was viable yet attenuated when compared to JFH1 [41]. In addition, the highly efficient J6CF p7 variant (GT2a) boosted virus production in the context of the homologous GT2a strain JFH1, but almost abrogated accumulation of infectious virions in the environment of the more distantly related GT 1b virus Con1/C3 [53]. On one hand, these data strongly suggest that p7 promotes virus particle production in a context-dependent and genotype-specific manner, most likely due to interactions with other viral factors. On the other hand, these results also suggest that p7 variants from divergent patient isolates (e.g., GT2a JFH1 and J6CF) differ with regard to their ability to sustain virus production suggesting that p7 modulates viral fitness. In agreement with this interpretation, using the trans-complementation system Brohm *et al.* provided evidence that a tyrosine residue close to the conserved di-basic motif of p7 is important for optimal virus production in the context of genotype 2a viruses [65].

Which viral factors, however, p7 cooperates with, is currently poorly defined. Nevertheless, first genetic evidence suggests that p7 may function in concert with NS2 or core: Yi *et al.* observed that an inter-genotypic virus chimera comprising H77 and JFH1 segments (GT1a-2a) fused at a position within NS2 acquired compensatory mutations residing in p7 and NS2 [26]. Moreover, when we mapped the optimal junction for Con1-JFH1 chimeras (GT1b-2a), a crossover downstream of the first TM-helix of NS2 was superior to a junction at the C-terminus of p7 [67]. Finally, core protein mutations lethal for virus assembly can be rescued by compensatory mutations within p7 [68]. Together these findings provide an important framework guiding further studies geared to reveal the mechanisms of p7 function. Notably, the discovery that vpu counteracts an interferon stimulated restriction factor (tetherin) that prevents release of infectious human immunodeficiency virus 1 (HIV-1), compared to the function of M2 described above, illustrates the diversity of mechanisms by which viroporins facilitate virus propagation.

## 3. P7-Based Antiviral Strategies

In theory, each step of the virus replication cycle and each cellular or viral factor involved in any of these steps is a possible drug target. The recent findings that p7 is essential for virus production combined with the knowledge that successful intervention with viral ion channel function is feasible, has nourished hopes that p7-based antiviral strategies can be implemented. Such strategies may rely on prevention of p7 oligomerization, its interaction with viral or host factors, or possibly blockage of the channel pore by small molecules. Although, robust *in vitro* assays for screening p7 inhibitors at high throughput level are still lacking, also whole life cycle screening systems may prove useful to identify molecules that interfere with p7 function. Several p7 inhibitors with at least confirmed antiviral activity in cell culture, and which primarily emerged from studies performed with viroporins from other viruses, have already been reported. These include amantadine, which inhibits the M2 channel of the influenza A virus [69–71], hexamethylene amiloride (HMA), an inhibitor of the HIV-1 vpu ion channel [72] and long-alkyl-chain iminosugar derivatives [38].

### 3.1. Amantadine

Amantadine is an organic compound also known as 1-aminoadamantane and inhibits the cell entry of influenza A viruses by interacting with the viroporin M2. It has proven efficacious in preventing influenza A virus infections although rapid emerge of drug-resistant variants have been reported. In fact utilization of amantadine and its derivative rimantadine for prophylaxis and treatment is no longer recommended in the USA due to high prevalence of drug-resistant virus (http://www.cdc.gov/flu/han011406.htm). Using solid-state NMR spectroscopy recently, two amantadine binding sites within the M2 ion channel were defined in the presence of phospholipid bilayers [73]. The high-affinity site, occupied by a single amantadine molecule, is located in the N-terminal channel lumen whereas a low-affinity binding site was observed on the C-terminal protein surface.

In 1997, Smith reported that amantadine treatment was effective in inducing sustained biochemical and virological response in patients with chronic hepatitis C who had previously failed to respond to interferon monotherapy [74]. However, other clinical studies failed to confirm a beneficial effect of monotherapy or combination treatment with IFN-α/ribavirin [75,76]. Further hopes that amantadine may inhibit HCV were fueled by the observation that amantadine prevents p7 ion channel activity in artificial membranes [33] and in a cell-based hemadsorption surrogate assay [48]. Leucine residues 51 through 57 of p7 were reported to be involved in a p7-amantadine interaction [77] and mutation of this leucine-rich region conveyed resistance of p7 to amantadine in a liposome-based ion channel activity assay [52]. However, using p7 peptides inserted into planar lipid bilayers, we observed inhibition of p7 ion channel activity only at high amantadine concentration [78]. Each study applied different p7 constructs with respect to peptide production, isolate/genotype, modification or mutation and different assay setups (planar lipid bilayers or liposome dye release) which might explain the discrepancies in amantadine inhibition.

In the HCV cell culture system amantadine at a dose of 50 μM did not affect RNA replication, particle production and infectivity of JFH1 and several JFH1-derived chimeras [78]. At higher concentrations a moderate inhibition of production of infectious HCV could be detected for some chimeric genomes suggesting a genotype-dependent inhibition by amantadine [79]. This genotype-dependent sensitivity has also been observed in p7 ion channel activity assays [79]. For example, JFH1-derived p7 is not blocked in a liposome-based ion channel assay and infectious particle production, whereas a genotype 3a p7 was susceptible to inhibition by amantadine in both systems. In addition, Griffin *et al.* found that inhibition profiles of p7 inhibitors in cell culture did not always reflect the profiles of different p7 ion channels activities *in vitro* [79]. Despite these partly discrepant findings, it is clear that the inhibition of HCV by amantadine is, at best, moderate. Moreover, the mostly disappointing results of clinical studies using amantadine in HCV infected patients do not support clinical use of this drug for HCV treatment. Nevertheless, amantadine may be a useful tool to study HCV assembly and p7 function *in vitro*.

### 3.2. Amilorides

Amiloride is a guanidinium group containing pyrazine derivative used in the management of hypertension and congestive heart failure. Similar to p7 the HIV-1 vpu-protein forms cation-selective ion channels *in vitro* and enhances the budding and release of virus particles [80]. In 2002, Ewart *et al.* demonstrated that amiloride derivatives block ion channel activity and enhancement of virus-like particle budding caused by HIV-1 vpu [72]. Modeling the binding of the amiloride derivative to vpu revealed that the inhibitor orientates along the channel axis with its alkyl ring pointed inside the pore and the hydrophilic serine-23 residue might be a favorable binding site [81]. Recently, p7 ion channel activity was also shown to be inhibited by hexamethylene amiloride [39]. In cell culture, however, cellular toxicity at drug concentrations required to achieve p7 inhibiton preclude a clear conclusion about the inhibitory effect of amilorides on HCV particle production in tissue culture [79]. To our knowledge clinical development of amilorides for HCV treatment is currently not pursued.

### 3.3. Iminosugar Derivatives

Iminosugars are monosaccharide mimics with the ring oxygen replaced by a nitrogen atom. They occur naturally in certain plants and microorganisms and cannot be metabolized by hydrolytic enzymes and the nitrogen lends itself well to adding various alkyl sidechains, which influence the compounds’ pharmacokinetic and toxicologic profiles [82], It was previously shown, applying the HCV surrogate model bovine diarrhea virus (BVDV) that glucose-derivatives deoxynojirimycin (DNJ)-containing iminosugars, such as *N*-*butyl*-DNJ (*N*B-DNJ) and *N*-*nonyl*-DNJ (*N*N-DNJ), are potent antivirals inhibiting ER α-glucosidases I and II [83]. These enzymes remove glucose residues from the high-mannose *N*-linked glycans attached to nascent glycoproteins and this processing step is essential for the subsequent interaction between the glycoproteins and ER chaperones calnexin and calreticulin [84]. Therefore, inhibition of α-glucosidases may interfere with proper folding and maturation of viral envelope glycoproteins and, consequently interfere with production of infectious particles. Importantly, long alkyl chain derivatives such as *N*N-DNJ and also the galactose-based *N*N-DGJ, a derivative of *N*N-DNJ which, due to its galactose-backbone, does not bind and interfere with alpha-glucosidases, also inhibit HCV p7 ion channel activity *in vitro*, whereas the short alkyl chain derivatives (*N*B-DGJ, *N*B-DNJ) are inactive in this assay [38]. Similar to data that have been described for BVDV, no effect of *N*N-DNJ and *N*N-DGJ on HCV RNA replication was observed [78,79]. Furthermore, none of the inhibitors affected viral protein synthesis and processing of the polyprotein precursor, corroborating the lack of cytotoxicity at the chosen concentrations. However, a dose-dependent reduction in the amount of infectious viruses of genotype 2a and 1b could be demonstrated [78,79]. Furthermore, a high dose of *N*N-DNJ successfully cleared HCV from cell cultures in the course of several consecutive cell culture passages [78]. This substantial antiviral effect may be attributable to the long alkyl side chain that confers an HCV p7 inhibitory effect. Thus, compounds combining a DNJ headgroup with a long alkyl side chain, such as *N*N-DNJ, may have two mechanisms of action: inhibition of ER-resident α-glucosidases and blockade of p7 ion channel activity. A derivative of *N*N-DNJ (UT-231B) has been administered in a clinical phase II study to previous non-responders to IFN-α-based therapy but has not proven significant antiviral efficacy [85]. In conclusion, iminosugar derivatives efficiently inhibited p7 ion channel activity *in vitro* and suppressed spread of HCV in cell culture making them attractive compounds for further investigations especially with respect to the mode of action.

### 3.4. BIT225

A recent study by the company Biotron identified a novel small molecule, termed BIT225, which represents a new class of p7 ion channel inhibitor [86]. BIT225, a substituted napthoyl guanidine, was identified after screening a compound library in a semi-quantitative, moderate throughput p7-based bacterial assay. The compound has a mid nanomolar IC_50_ against BVDV and shows antiviral synergy with IFN-α, ribavirin and NS5B polymerase inhibitors. Furthermore, BIT225 was able to inhibit p7 ion channel function in planar lipid bilayers and has successfully completed a phase Ia dose escalating, single dose safety trial, in healthy volunteers and a phase Ib/IIa trial to evaluate the safety and pharmacokinetics of repeated dosing for selected doses of BIT225 in HCV-infected persons. A modest, but statistically significant drop in patient’s viral load was detected over seven days of dosing [86]. The effect of BIT225 in the HCV cell culture system has not yet been described.

## 4. Conclusion and Outlook

The recent development and application of HCV cell culture systems has firmly established that p7 is essential for production of infectious virus progeny. In parallel, *in vitro* studies using artificial membranes have highlighted the ability of p7 to oligomerize and to conduct ions in a cation-selective fashion. However, the conceptual, mechanistic and experimental link, as to how the p7 ion channel function contributes to production of infectious particles, is still incomplete. Certainly, manipulation of the pH of the cellular secretory compartment by p7 may facilitate maturation, folding and transport, of essential viral assembly factors and thus contribute to production of infectious particles. While in this model p7 is an “M2-like” protein, evidence for proton channel activity in artificial membranes or, more importantly, within virus producing cells has not been provided. At least the localization of p7 primarily at the ER is in principle compatible with this model. At this point, however, alternative models cannot be dismissed. The recent finding that vpu facilitates release of HIV-1 particles by interfering with tetherin, is just one example that illustrates the diversity of possible mechanisms. In this case, it is unclear if and how vpu-dependent changes of membrane permeability contribute to interference with tetherin.

Further dissection of specific, and possibly unique, properties of p7 is likely to define how this protein operates in the course of HCV assembly. The findings that p7 acts in an isolate-specific fashion, possibly by interacting with other viral factors, and the observation that p7 from different viral strains sustain virus production with divergent efficiency, are important leads that may be translated into mechanistic insights in the future. Certainly, identification of viral and host proteins that interact with p7 should help to characterize its mode of action. Moreover, finding answers to the question how p7 conducts ions, which ions are transported, and at which location within cells p7 modulates the ion milieu, will be critical. At present, the relevance of p7 ion channel function for virus production is mainly based on intriguing correlations: mutations that abrogate ion channeling in artificial membranes also interfere with production of infectious particles. In addition some small molecules that ablate channeling *in vitro* have been shown to moderately reduce virus production. However, direct proof that these molecules specifically act via p7, thus preventing virus production, is still elusive. For M2 of influenza A, mutations conferring resistance to amantadine map to M2. In addition, these mutations confer resistance of the virus to amantadine and at the same time to the M2 ion channel in cell based channel assays. In that very same sense the emerging novel compounds which presumably inhibit p7 may be instrumental to firmly establish the relevance of its ion channel function. In the long run, some of these molecules characterized by basic science at the laboratory bench, may progress to the bedside, thus expanding therapeutic options for treatment of hepatitis C.
